# Isothiocyanate-Rich Moringa Seed Extract Activates SKN-1/Nrf2 Pathway in *Caenorhabditis elegans*

**DOI:** 10.3390/ijms252010917

**Published:** 2024-10-10

**Authors:** Renalison Farias-Pereira, Pierre Camayoc, Ilya Raskin

**Affiliations:** 1 Department of Biological Sciences, The Dorothy and George Hennings College of Science, Mathematics and Technology, Kean University, Union, NJ 07083, USA; 2 Department of Plant Biology, School of Environmental and Biological Sciences, Rutgers, The State University of New Jersey, New Brunswick, NJ 08901, USA

**Keywords:** medicinal plant, oxidative stress, toxicity

## Abstract

*Moringa oleifera* is a tropical tree that has its leaves, fruits, and seeds used as medicine and food. A standardized hydroalcoholic moringa seed extract (MSE) contains up to 40% of an isothiocyanate (MIC-1; moringin), a phytochemical known to have antioxidant and anti-inflammatory properties. Animal studies suggest that MSE may help with diseases, such as edema, colitis, obesity, and diabetes. In vitro studies have shown that MIC-1 activates the Nrf2 pathway, involved in detoxification and antioxidant pathways. To broaden the understanding of the molecular pathways regulated by MSE, we hypothesized that MSE improves the health span in *Caenorhabditis elegans* by activating the Nrf2 homolog (SKN-1). Our whole RNA-seq data showed that MSE at 0.1 mg/mL (100 µM MIC-1) regulated the expression of a total of 1555 genes, including genes related to *C. elegans* cuticle, molting cycle, and glutathione metabolism. MSE upregulated several glutathione S transferases (GST), involved in the detoxification of xenobiotics, and other SKN-1 downstream targets. MSE and MIC-1 upregulate *skn-1* expression and induce SKN-1 nuclear translocation, suggesting that they activate the SKN-1/Nrf2 pathway. Moreover, the regulation of glutathione metabolism is likely dependent on the SKN-1 pathway, as the *gst-4* upregulation by MSE was inhibited in *skn-1* knockout mutant. However, MSE decreased survivability and delayed growth rate, while purified MIC-1 increased the lifespan of *C. elegans*. This study shows that MIC-1 is responsible for SKN-1/Nrf2 activation by MSE; however, components other than MIC-1 within MSE likely cause detrimental effects in *C. elegans*.

## 1. Introduction

*Moringa oleifera*, commonly known as the drumstick tree, is native to South Asia and has been cultivated for traditional medicine uses [1]. Known for its rapid growth and ability to thrive in harsh conditions, moringa has been used for a variety of health benefits as it is rich in nutrients and bioactive compounds that help to decrease blood sugar and improve immunity against various diseases. Moringa seeds contain known antioxidant compounds, such as flavonoids and isothiocyanates [2,3,4,5]. Isothiocyanates are indirect antioxidants as they induce the nuclear factor erythroid 2-related factor 2 (Nrf2), a transcription factor that regulates the phase II detoxifying and antioxidant enzymes. Moringa isothiocyanate-1 (MIC-1; moringin) has antioxidant, immunomodulating, and anti-inflammatory responses at the gene expression levels through the induction of the Nrf2 pathway and the inhibition of the nuclear factor kappa-light-chain-enhancer of activated B cells translocation in muscle cells [6,7]. The upregulation of enzymes, such as glutathione S transferases (GST), by MIC-1 prevents cell damage through their reaction to reduced glutathione, to xenobiotics, or to reactive oxygen species [3,8].

It is likely that moringa seed extract (MSE) and MIC-1 share similar biological properties since MIC-1 is MSE’s main bioactive compound (up to 40% w/w). Indeed, studies have shown that MSE also possesses antioxidant and anti-inflammatory properties. In rodent studies, MSE has been shown to mitigate inflammatory-related diseases and symptoms, such as colitis, diabetes, and edema [3,5,8]. MSE upregulates the phase II detoxifying enzymes NQO and GST in the murine gastrointestinal system at transcript levels [3,8]. However, whether isothiocyanate-rich MSE may attenuate oxidative stress-related human diseases via the Nrf2 pathway is unknown.

The nematode *Caenorhabditis elegans* is an animal model used to elucidate the molecular pathways regulated by plant extracts and bioactive compounds since many of its genes are homologs to human disease-related genes [9]. Moreover, studies on the pharmacological effects of bioactive compounds on lifespan and molecular targets in *C. elegans* are possible because of its short life cycle, body transparency, and sequenced and annotated genome. For example, moringa leaf extract increased lifespan and stresses resistance of *C. elegans* [10,11]. However, the cellular mechanism of this effect is not fully known. Moreover, *C. elegans* is used to investigate the toxic effects of xenobiotics, including pharmaceuticals and food contaminants [12]. Some of the toxicity parameters observed in *C. elegans* are the shortened lifespan and delayed growth rate, in addition to the induction of phase I-III detoxification enzymes [12,13]. Since the *C. elegans* SKN-1 is a homolog to the human Nrf2 regulating phase II detoxification downstream genes, such as GST [14,15,16,17,18,19], this study aimed to broaden the understanding of MSE and MIC-1’s molecular targets and their effects on *C. elegans* life span and gene expression. We hypothesized that MSE activates the SKN-1/Nrf2 pathway, potentially leading to either beneficial or detrimental effects on health span of nematodes.

## 2. Results

**Figure 1:**
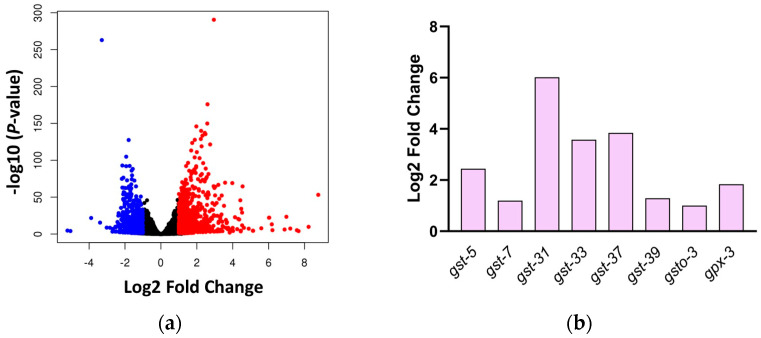
Transcriptomics analyses of moringa seed extract (MSE)-treated nematodes. Nematodes at the L1 stage were treated with 0.1 mg/mL moringa seed extract (MSE) for 2 days before performing differential gene expression analyses compared to untreated control. (**a**) A total of 935 genes were upregulated (red) and 620 genes were downregulated (blue) by MSE. Black dots represent genes not regulated by MSE. (**b**) Glutathione-related genes significantly upregulated by MSE.

We used a non-targeted transcriptomics approach to identify MSE’s potential molecular targets and pathways in nematodes treated with 0.1 mg/mL MSE for 2 days before RNA-sequencing. A total of 1555 genes were found to be significantly regulated by MSE, of which 935 genes were upregulated and 620 genes were downregulated (Figure 1a). Among the most significantly repressed or induced genes by MSE is a glutathione S transferase (*gst-5*), a predicted target of the SKN-1 pathway (Supplementary Figure S1; Supplementary File S1). Consistently, MSE upregulated other glutathione-related genes (*gst-7*, *gst-31*, *gst-33*, *gst-37*, *gst-39*, *gsto-3*, and *gpx-3*) (Figure 1b). The upregulation of glutathione-related genes by MSE suggests an activated phase II detoxification, but these results also show that MSE had an impact on the overall nematodes’ cell physiology. Other pathways regulated by MSE also include extracellular matrix organization, cuticle, molting cycle, negative regulation of endopeptidase activity, and eggshell formation in *C. elegans* (Supplementary Figure S2; Supplementary File S2).

**Figure 2:**
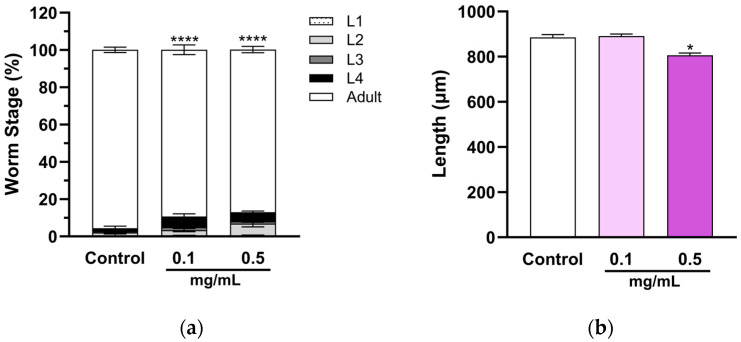
Moringa seed extract (MSE) delays development of nematodes. *C. elegans* were treated with MSE for 3 days from synchronized L1 stage. (**a**) Growth rate: the developmental stages of MSE-treated nematodes were observed and compared to the control using the chi-square test (**** *p* < 0.0001, *n* = 438–662). (**b**) Length: the size of *C. elegans* treated with MSE was measured using ImageJ 1.53t. MSE-treated nematodes were compared to the control group using *t*-test (* *p* < 0.05, *n* = 30). Mean ± SEM.

Since multiple pathways were affected by MSE in *C. elegans*, we evaluated the MSE’s effects on the development of nematodes by treating them with MSE (0.1–0.5 mg/mL) from L1 stage for 3 days. The developmental stages were observed in a microscope and photos were taken for length measurements. MSE delayed the development of nematodes in comparison to the control group (Figure 2a). Consistently, the higher concentrations of MSE reduced the nematodes’ length (Figure 2b), suggesting that MSE has a dose-dependent effect on nematode development, potentially caused by the changes in the molting cycle and cuticle.

**Figure 3:**
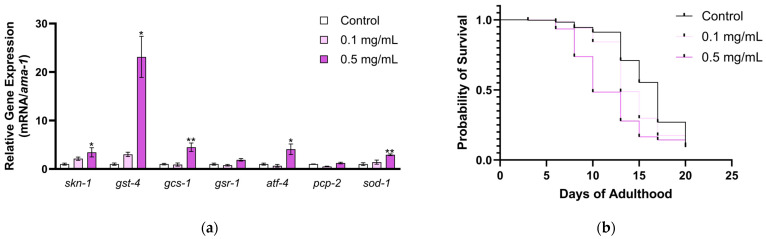
Moringa seed extract (MSE) regulates SKN-1-related genes, increasing the mortality of *C. elegans*. (**a**) RT-qPCR: Nematodes were treated with MSE from the L1 stage for 2 days. Power SYBR Green PCR master mix was used as the detection method, and data are shown as relative to the control and housekeeping gene (*ama-1*). Ordinary one-way ANOVA or the Kruskal–Wallis test (only for *gst-4* dataset) were performed to obtain *p*-values (* *p* < 0.05, or ** *p* < 0.01) across MSE and control groups. Mean ± SEM (*n* = 3). (**b**) Lifespan: Nematodes were treated with MSE throughout their adulthood. MSE, both at 0.1 and 0.5 mg/mL, decreased the lifespan of nematodes with *p* < 0.0001, obtained via log-rank (Mantel–Cox) test, compared to the control (*n* = 293–349).

Since MSE upregulated glutathione-related genes in *C. elegans*, we further evaluated MSE’s effects on the SKN-1 pathway; we chose some of the most prominent SKN-1 downstream targets in *C. elegans*. Nematodes were treated with 0.1–0.5 mg/mL MSE from L1 stage; then, the expression of SKN-1-related genes were measured by RT-qPCR. MSE at 0.5 mg/mL significantly upregulated *skn-1* (*p* = 0.0479), *gst-4* (*p* = 0.0146), *gcs-1* (*p* = 0.0070), *atf-4* (*p* = 0.0289), and *sod-1* (*p* = 0.0082) at the transcript level when compared to the control (Figure 3a). The SKN-1 pathway is also relevant for the *C. elegans* lifespan; thus, we further evaluated the toxic effects of MSE on nematodes. Nematodes were treated with MSE at 0.1–0.5 mg/mL throughout their adulthood. MSE-treated nematodes had a shorter lifespan in comparison to the control group (Figure 3b). Treatment with MSE at 0.1 mg/mL decreased the median lifespan from 17 to 13 days in comparison to the control (*p* < 0.0001), while MSE at 0.5 mg/mL decreased the median lifespan from 17 to 10 days (*p* < 0.0001). Although MSE activates the SKN-1 pathway, MSE increased the mortality of *C. elegans*.

**Figure 4:**
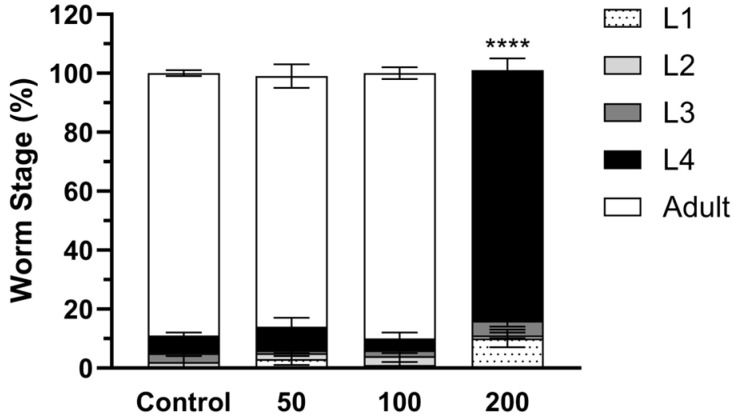
Moringa isothiocyanate-1 (MIC-1) delays the development of nematodes. *C. elegans* were treated with MIC-1 for 3 days from the synchronized L1 stage. The developmental stages of MIC-1-treated nematodes were observed and compared to the control (0.1% DMSO-treated nematodes) using the chi-square test (**** *p* < 0.0001, *n* = 100–121).

To test the hypothesis that MIC-1 is responsible for the effects of MSE, we evaluated the effects of MIC-1, the main antioxidant and anti-inflammatory component of MSE, in *C. elegans*. The growth rate of nematodes was assessed after treatment with 50–200 µM MIC-1 or vehicle control (0.1% DMSO), starting at the L1 stage for 3 days. MIC-1, only at 200 µM, delayed the growth rate of nematodes, while there was no statistical difference between 50–100 µM MIC-1 in comparison to the 0.1% DMSO-treated nematodes (Figure 4).

**Figure 5:**
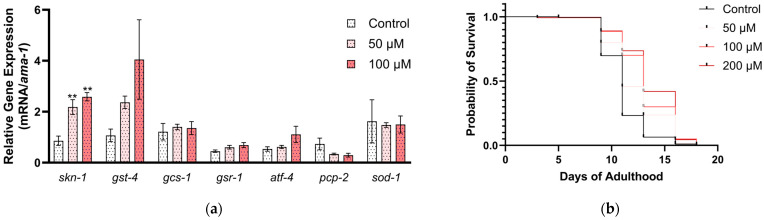
Moringa isothiocyanate-1 (MIC-1) regulates SKN-1-related genes, increasing the lifespan of *C. elegans*. (**a**) RT-qPCR: Nematodes were treated with MIC-1 from the L1 stage for 2 days. Power SYBR Green PCR master mix was used as the detection method, and data are shown as relative to the control and housekeeping gene (*ama-1*). Ordinary one-way ANOVA was performed to obtain *p*-values (** *p* < 0.01) across treatment and control groups (0.1% DMSO-treated group). Mean ± SEM (*n* = 3). (**b**) Lifespan: Nematodes were treated with MIC-1 throughout their adulthood (*n* = 136–185). The log-rank (Mantel–Cox) test was used for comparison between control and treatments; *p* < 0.0001 for all MIC-1 treatments compared to the control (0.1% DMSO).

We additionally tested MIC-1’s impact on the expression of SKN-1-related genes. The SKN-1 downstream target genes, including glutathione-related genes, were not statistically changed by MIC-1 in *C. elegans* (Figure 5a), which suggests that higher MIC-1 doses are needed to observe the upregulation of SKN-1 downstream target genes; 0.5 mg/mL MSE contains approximately 500 µM MIC-1. Interestingly, the expression of *skn-1* was upregulated by lower concentrations of MIC-1 at 50 µM (2.2-fold; *p* = 0.0098) and 100 µM (2.6-fold; *p* = 0.0027).

The effects of MIC-1 on the *C. elegans* lifespan were also investigated to determine if MIC-1 is responsible for the increased mortality in the MSE-treated nematodes. Unlike MSE, which increased mortality, MIC-1 increased the lifespan of *C. elegans* (*p* < 0.0001; Figure 5b). MIC-1 at 100 and 200 µM increased median survival from 11 to 13 days in comparison to the vehicle control.

**Figure 6:**
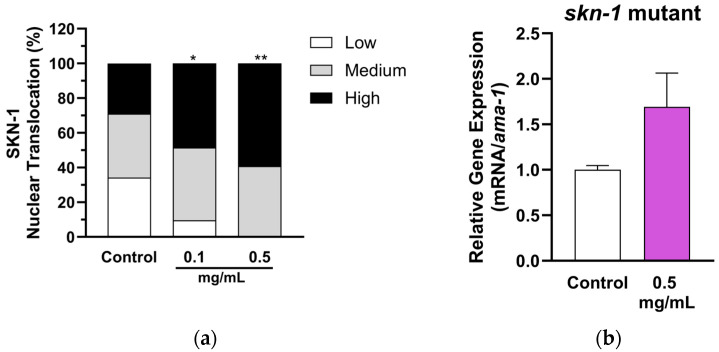
Moringa seed extract (MSE) upregulates glutathione transferase by inducing SKN-1 nuclear translocation. (**a**) Nuclear translocation of SKN-1::GFP induced by MSE. Images were captured using the Leica DM4 B Fluorescence LED Microscope. Nematodes were observed and classified into different levels of nuclear translocation levels. MSE at 0.1–0.5 mg/mL induced nuclear translocation of SKN-1 compared to the control group using the chi-square test. * *p* < 0.05 or ** *p* < 0.01 (*n* = 22–38). (**b**) MSE did not regulate *gst-4* expression in *skn-1* mutant (QV225 nematode strain). Relative gene expression of *gst-4* was normalized using the Ct values of the control group and housekeeping gene (*ama-1*). Mean ± SEM (*n* = 3).

We further tested whether MSE induces SKN-1 nuclear translocations. The SKN-1::GFP mutants were treated with MSE for two days before observations using a fluorescence microscope. MSE increased SKN-1 nuclear translocation compared to the control (Figure 6a). We also tested whether the upregulation of *gst-4* by MSE was dependent on *skn-1* by measuring the gene expression in *skn-1* knockout mutant. MSE at 0.5 mg/mL was not able to significantly upregulate *gst-4* in *skn-1* mutant (Figure 6b), suggesting that MSE’s effects on glutathione metabolism is dependent on the SKN-1 pathway in *C. elegans*.

## 3. Discussion

This is the first study that investigated whether MSE activates the Nrf2 pathway in vivo by exploring the transcriptional effects of MSE in *C. elegans*, a multicellular invertebrate organism whose Nrf2 homolog is called SKN-1. In summary, a low concentration of MSE (0.1 mg/mL, equivalent to approximately 100 µM MIC-1) affected the expression of multiple genes in *C. elegans*, including the upregulation of multiple glutathione S transferases (GST). Meanwhile, a higher concentration of MSE (0.5 mg/mL; 500 µM MIC-1) upregulated *gst-4* dependently on *skn-1*, suggesting that the upregulation of GST is achieved via the SKN-1/Nrf2 pathway. However, SKN-1 activation by MSE is likely associated with the potentially toxic effects of MSE in *C. elegans*; MSE reduced both growth rate and lifespan. These toxic effects may be caused by other compounds present in MSE. On the other hand, purified MIC-1 (50–200 µM), an active antioxidant and anti-inflammatory component of MSE present at up to 40% w/w, had less effect on the growth rate and increased the lifespan of nematodes. It is likely that MIC-1’s effects are mediated through the SKN-1 pathway, as MIC-1 also upregulates *skn-1*. Since both MSE and MIC-1 activated the SKN-1 pathway in *C. elegans*, MIC-1 is likely the key compound responsible for the activation of the SKN-1/Nrf2 pathway by MSE.

SKN-1/Nrf2 protects cells against stressors, such as xenobiotics and oxidative stress [19]. For example, SKN-1/Nrf2, when in the cell’s nucleus, induces GST expression, a phase II detoxification enzyme that is responsible for the conjugation of glutamate to xenobiotics, and superoxide dismutase (SOD), an antioxidant enzyme that counteracts the effects of oxidative stress. Our results are consistent with the known effects of nuclear translocation of SKN-1/Nrf2 that controls the expression of detoxification and antioxidant genes [12], as well as with previous studies on MSE/MIC-1’s effects on antioxidant targets [3,6,8]. This study is the first to show that MSE activates the Nrf2 pathway in vivo due to the presence of MIC-1. Isothiocyanates, such as MIC-1, bind cysteines residues in Kelch-like ECH-associated protein 1 (Keap1), releasing Nrf2 to the cell’s nucleus [6,14]. However, the SKN-1 activation may be mediated by other pathways, such as ubiquitin-mediated proteolysis and the insulin pathway, since *C. elegans* does not have a Keap1 ortholog [14].

The strong effects of MSE on glutathione metabolism may partially explain its toxic effects in *C. elegans*. Glutathione is involved in the protection of cells acting in redox reactions and coupling with xenobiotics for their elimination [20]. Consistently, others have shown that MSE and MIC-1 upregulate GST and GCL in mammalian models [8]. In *C. elegans*, the upregulation of GST-4 and GCS-1 may indicate oxidative stress caused by hydrogen peroxide and other reactive oxygen species [21]. Therefore, a dose of MSE may cause a redox imbalance due to excessive dietary antioxidants shortening the lifespan of *C. elegans* [22]. Others have shown that an allyl isothiocyanate also induces GST-4 expression dependently on SKN-1 [23], suggesting that MIC-1 may be responsible for MSE’s effects on glutathione metabolism. However, further studies are needed to elucidate the effects of MIC-1 on glutathione metabolism and its potential link to the survival rate and stress responses.

SKN-1/Nrf2 activation is related to an increased survival rate of nematodes, especially under stresses [14,15,17,18,19]. It is believed that SKN-1’s effects on survival is mediated by reduced insulin signaling led by the Forkhead box O transcription factor DAF-16 in *C. elegans* [14]. Plant extracts and compounds, including isothiocyanates, increase the lifespan of nematodes by reducing the insulin signaling pathway via DAF-16 [10,11,24,25]. For example, sulforaphane, a broccoli isothiocyanate known to activate the Nrf2 pathway, increases the lifespan of *C. elegans* via the insulin signaling pathway [24,25]. Similarly, moringa leaf extracts increased the lifespan via the DAF-16 pathway [10,11]. Although in this study, MSE did not increase the lifespan of nematodes, it was previously observed that MSE reduced glucose levels, which is associated with increased antioxidant markers in high-fat-diet-fed mice [3]. However, purified MIC-1, up to 200 µM, increased the lifespan of nematodes, but its effects on the insulin signaling pathway are yet to be determined.

In addition to MIC-1, MSE contains other plant secondary metabolites, such as procyanidins, glucosinolates, and thiocarbamates, which may contribute for the effects of MSE in *C. elegans* [5]. For example, a procyanidin-rich leaf extract from *Combretum mucronatum* have antinematicidal properties by regulating several detoxification enzymes, such as *gst-24* in *C. elegans* [26]. Although glucosinolates are the precursor of isothiocyanates, they can also be converted to potential nematicides, such as carbamates and thiocarbamates (e.g., niazimicin) [5,13,27]. The lethality of allyl glucosinolates was dependent on the presence of glucosidases, which are responsible for the formation of glucosinolates derivatives [28,29]. The presence of glucosidases and glucosinolates within MSE could explain the reduced lifespan in *C. elegans*. Moreover, the excessive intake of macronutrients provided by MSE may be responsible for the reduced lifespan and development in *C. elegans* [30]; MSE contains about 18% of protein, 23% of carbohydrates, and 3% of fat [5]. In rats, the no-observed-adverse-effect level for MSE daily oral ingestion is 257 mg/kg bw, equivalent to 100 mg of MIC-1 [31]. Higher doses of MSE, up to 2571 mg/kg bw, caused some changes in toxicological parameters (vocalization, respiratory pattern, body weight and food consumption) during 14 days treatment [31].

In conclusion, it is likely that the MSE reduces lifespan by disbalancing overall cell homeostasis, and the SKN-1 activation was insufficient to mitigate the xenobiotic stress. Although *C. elegans* can be used as a toxicological model, the translation of nematode’s xenobiotics response to humans is still limited [12]. MSE’s effects on other pathways, such as extracellular matrix organization, endopeptidase activity, cuticle, and molting, may also be related to overall toxicity and reduced growth rate of nematodes [32]. This study, including the non-targeted transcriptomics data, provides potential mechanisms insights into both MIC-1 and MSE’s health effects. For example, given SKN-1’s role in lipid metabolism and MSE’s downregulation of *fat-7* and *ech-7*, MSE or MIC-1 could be a potential fat-lowering agent, as are other plant-derived compounds [33]. Further studies may need to elucidate the SKN-1’s roles in these other pathways and address which other components within MSE contribute to its overall activity in *C. elegans*.

## 4. Materials and Methods

### 4.1. Plant Materials

Moringa seed extract (MSE) was created by the incubation of powdered seeds with water and ethanol, followed by the filtration of its solids, as previously described [5]. Powdered moringa seeds were incubated in water for 2 h at 37 °C; then, 95% ethanol was added to the solution. Solids and solvents from the extract were removed via vacuum filtration and rotary evaporation, respectively. Then, MSE was freeze-dried to powder and stored at −20 °C until its use. We further confirmed that MSE contained 33% of moringa isothiocyanate-1 (MIC-1) via LC-MS (Supplementary Figure S3). MIC-1 was purified via fraction centrifuge liquid chromatography using hexane, methanol, water, and ethyl acetate [5]. Stock solution of MSE at 2 mg/mL were freshly made in nematode liquid media (S-complete) and filtered in 0.22 µm sterile syringe filters, while MIC-1 (200 mM) was dissolved in dimethyl sulfoxide (DMSO) and stored at −20 °C until its use. We further confirmed that MSE delivers MIC-1 to nematodes by measuring MIC-1 levels via LC-MS in *C. elegans* after MSE treatment (Supplementary Figure S4).

### 4.2. Nematodes Culture

Nematodes were grown in solid growth media before eggs synchronization by using bleaching method [9]. Treatments were performed in S-complete with ampicillin (100 µg/mL), carbenicillin (50 µg/mL), and *Escherichia coli* OP50 as a food source. A total of 120 µM fluorodeoxyuridine was added to the media to stop the eggs from hatching when treatment started at the L4 stage or adulthood. Nematodes were cultured at 20 °C in an incubator with a 12 h light/dark cycle. *Caenorhabditis* Genetics Center (Minneapolis, MN, USA) provided the *E. coli* OP50 and *C. elegans* strains: the wild isolate N2, the mutants LD1 [*skn-1b/c::GFP* + *rol-6(su1006)*], and QV225 [*skn-1(zj15) IV*].

### 4.3. RNA Sequecing

Wild-type N2 nematodes were treated with 0.1 mg/mL MSE from the L1 stage for 2 days. The chosen dose was based on our preliminary data and a previous study with moringa leaf extracts in *C. elegans* [10]. After treatment, nematodes were collected in tubes and sent to Azenta Inc. (South Plainfield, NJ, USA) for RNA sequencing analyses. Samples were read approximately 20 million times on an Illumina next-generation sequencing platform (2 × 150 bp). The normalized gene hit counts were used to identify differentially expressed genes between control samples (N2) and treatment (MSE) using DESeq2 (version 1.44.0), the Benjamini–Hochberg adjusted *p*-value < 0.05, and fold change > 1. Moreover, gene ontology was performed by using GeneSCF v.1.1.-p2.

### 4.4. Developmental and Survival Assays

L1-stage nematodes were treated with MSE (0.1–1 mg/mL) or MIC-1 (50–200 µM) at 20 °C for three days. MSE-treated nematodes were compared to nematodes without any treatment (control), while MIC-1-treated nematodes were compared to 0.1% DMSO (vehicle control). The developmental stages of nematodes were identified in a microscope and data were represented as a percentage of the total population. The length of nematodes was measured in ImageJ, with pictures taken at 40× total magnification.

For survival rates, nematodes were cultured in a 96-well plate with approximately 20 worms/well in 200 µL of treatment solutions. The total number—alive or dead—nematodes was counted until all the nematodes had died. Data are represented as survival probabilities by dividing the number of alive nematodes by the total number.

### 4.5. qPCR

RNA was extracted using Tri-Xtract™ reagent followed by isopropanol precipitation, as recommended by the manufacturer (G Biosciences, Geno Technology Inc., St. Louis, MO, USA). RNA samples were used to synthesize cDNA by using UltraScript™ cDNA synthesis kit (PCR Biosystems, Wayne, PA, USA). Gene expression assays were performed using Step One Plus real-time PCR system and Power SYBR™ Green PCR master mix (Applied Biosystems, Foster City, CA, USA). The *ama-1* expression was used as an internal control to calculate gene expression via the delta–delta Ct method. The primer sequences used in this study are described in Supplementary Table S1.

### 4.6. SKN-1 Imaging

MSE-treated LD1 nematodes, SKN-1::GFP strain mutants, were immobilized with 10 mM sodium azide then transferred to a flat pad of 3% agarose in a microscope slide before covering with a cover slip. Images were captured using the Leica DFC7000 T camera on the Leica DM4 B Fluorescence LED Microscope, with the Leica Application Suite X version 1.4.7 software (Leica Microsystems Inc., Deerfield, IL, USA) controlling the camera settings and capturing the images. Nuclear translocation was scored as low, medium, and high, as described in Supplementary Figure S5.

### 4.7. Statistical Analysis

Data are represented as the mean with the standard error of the mean (SEM). *p*-value < 0.05 was used to indicate statistical significance. Statistical tests were performed in Prism 10 (GraphPad Software LLC, Boston, MA, USA), considering normality and data distribution as specified in the figure legends.

## Data Availability

The original contributions presented in the study are included in the article/Supplementary Materials, further inquiries can be directed to the corresponding author.

## Supplementary Materials

The supporting information can be downloaded at https://www.mdpi.com/article/10.3390/ijms252010917/s1.
