# Supplementary material for: Isothiocyanate-Rich Moringa Seed Extract Activates SKN-1/Nrf2 Pathway in Caenorhabditis elegans
Source: Int J Mol Sci. 2024 Oct 10;25(20):10917. doi: 10.3390/ijms252010917 (PMC11507030; doi:10.3390/ijms252010917)
Supplement: Supplementary file 1 [file ijms-25-10917-s001.zip › Supp Material.pdf]

Isothiocyanate-rich moringa seed extract activates SKN-1/Nrf2 pathway in *Caenorhabditis elegans*

Supplementary Materials

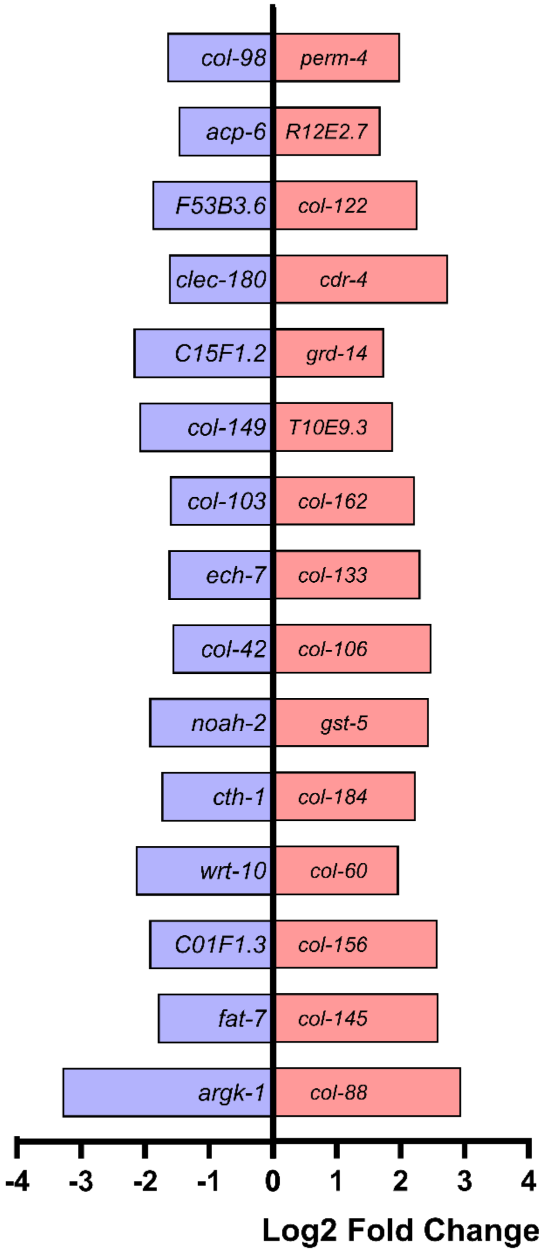

Supp. Fig. 1. Transcriptomics analyses of moringa seed extract (MSE)-treated nematodes. The top 15 most significant genes upregulated (red) or downregulated (blue) by MSE.

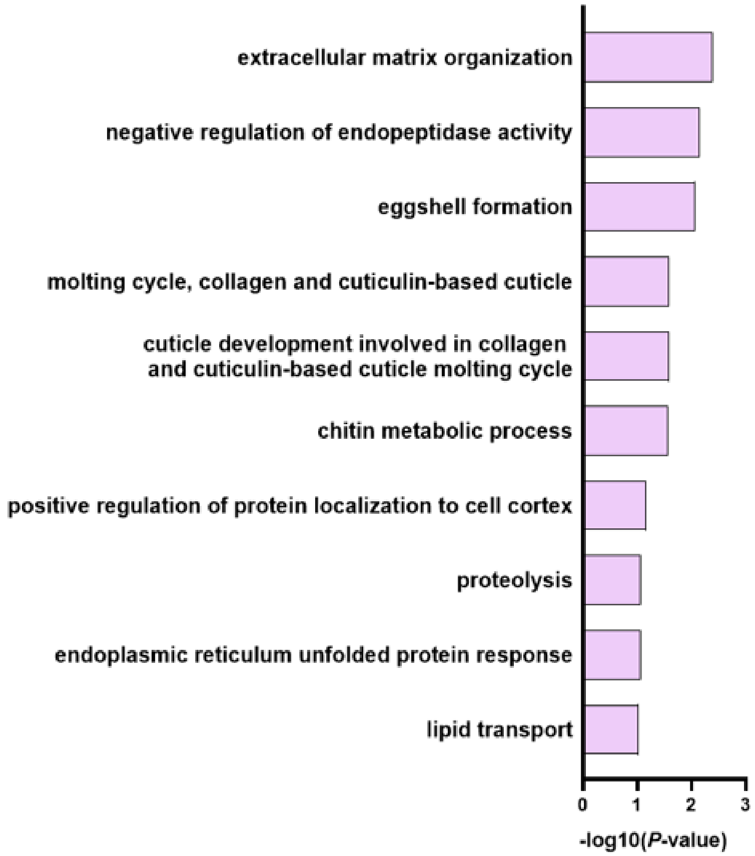

Supp. Fig. 2. Gene ontology analysis of moringa seed extract (MSE)-treated nematodes. Statistically significant set of genes were clustered based on their biological processes using the software GeneSCF v.1.1-p2. The figure shows the top 10 most regulated pathways by MSE in compared to the control.

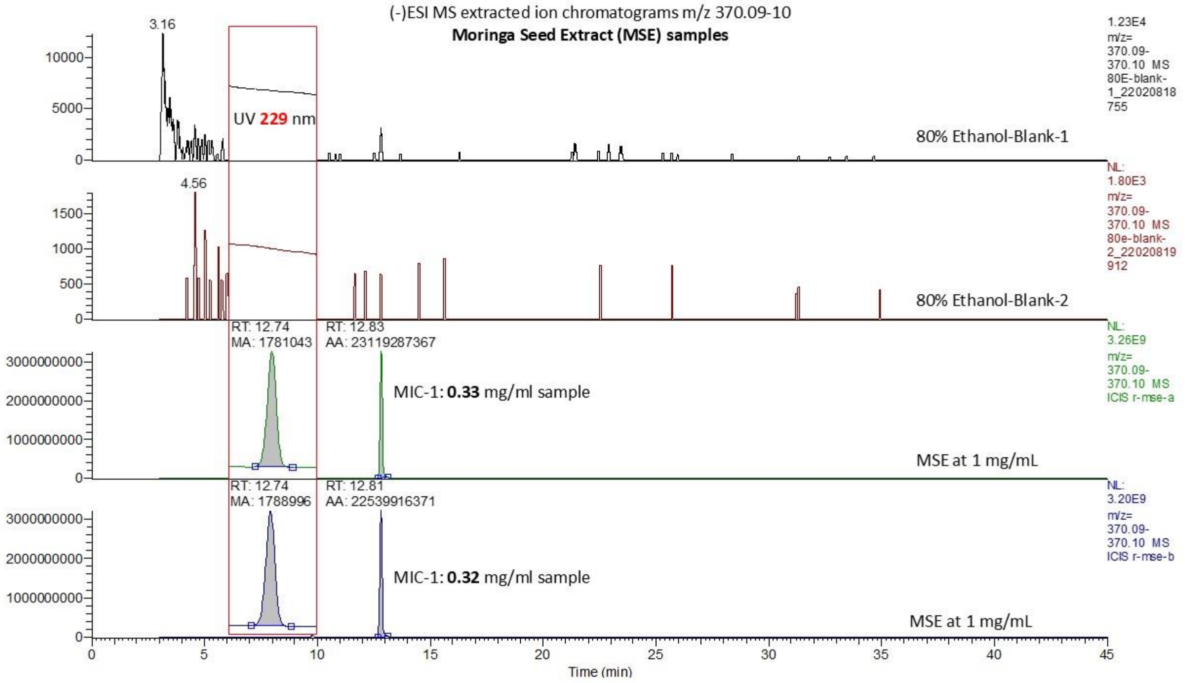

Supp. Fig. 3. Moringa seed extract (MSE) contains 33% of moringa isothiocyanate-1 (MIC-1). MSE was diluted in 80% ethanol, used as blank, thus solution was filtered using a syringe 0.2 µm filter. Liquid-chromatography mass spectrometry (LC-MS) was performed following previous described method [5]. Peaks of MIC-1 were found at retention time (RT) of 12.81-12.83 min, UV scan at 229 nm, and mass-to-charge ratio (m/z) of 370.09-370.10 in negative mode electrospray ionization (ESI). Absolute abundance was converted into concentration (mg/mL) using a standard curve with purified MIC-1.

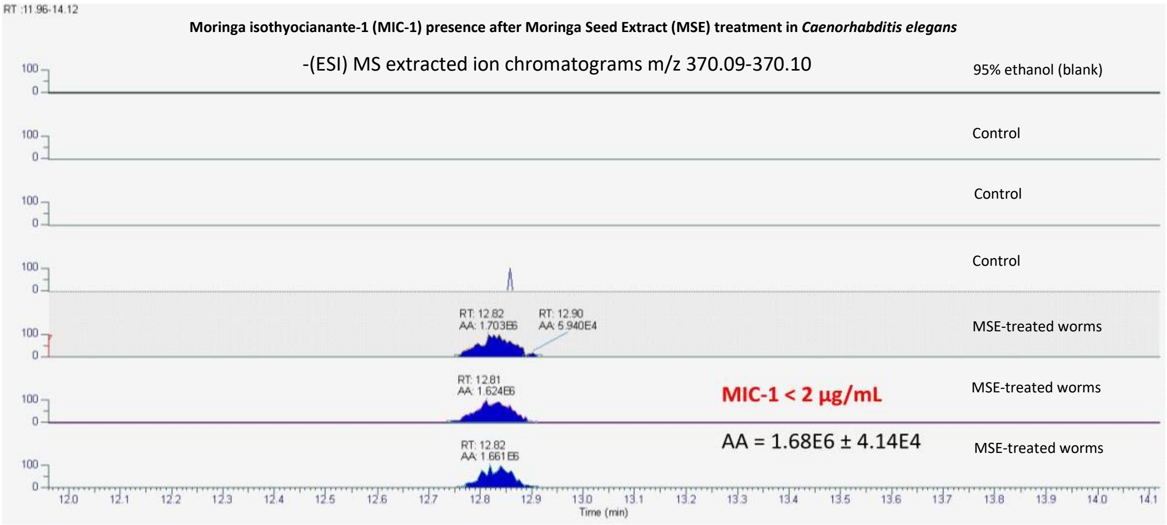

Supp. Fig. 4. Moringa isothiocyanate-1 (MIC-1) uptake into nematodes after 0.5 mg/mL of moringa seed extract (MSE). Nematodes at L4 stage were treated with or without 0.5 mg/mL MSE for 2 days, then collected, and washed in water before metabolites extraction. Nematodes were macerated with a pestle for 5 min, then sonicated for 30 min in 95% ethanol. Before evaporation of solvent, solution was filtered using a 0.45 µm syringe filter. Metabolite extracts were redissolved in 95% ethanol to 1 mg/mL before LC-MS run as Supp. Fig. 1. The most abundant peaks were identified as MIC-1, however at concentration below 2 µg/mL. It was estimated that 0.4% MIC-1 is found in *C. elegans* after 0.5 mg/mL MSE treatment, however higher doses are needed to increase accuracy of this method.

Low **Medium** **High**

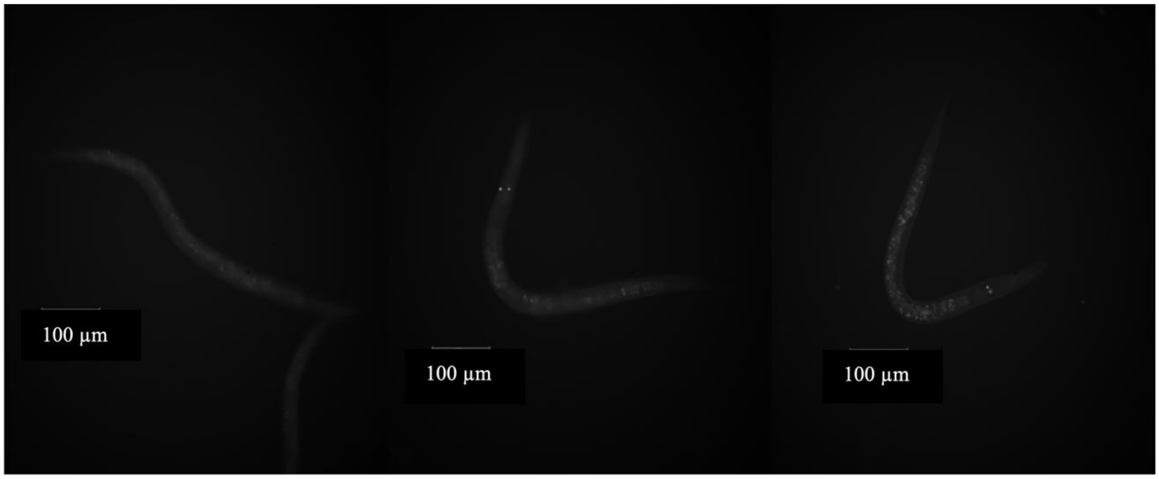

Supp. Fig. 5. Criteria for nuclear translocation of SKN-1::GFP categories. The nuclear translocation patterns of SKN-1::GFP in *C. elegans* were categorized as low, medium, and high based on fluorescence visibility across different body regions. Low translocation was observed in worms with almost no visible nuclear translocation. Medium translocation refers to worms with nuclear translocation in only parts of the body. High translocation is noted in worms with nuclear translocation visible throughout the entire body.

Supp. Table 1. Primers used for qPCR.

Name	Primer	Sequence
BZIP domain-containing protein (*skn-1*)	Forward	GAGCCTCAATCTGGAGCTTATC
	Reverse	TGGTAGAGGATGCCAGAAGA
DNA-directed RNA polymerase II subunit RPB1 (*ama-1*, housekeeping gene)	Forward	GTACGATCCTGACCCAAAGAAC
	Reverse	ATGGAGAGGTACGCGATAGA
Superoxide dismutase (*sod-1*)	Forward	CACGTAGGCGATCTAGGAAATG
	Reverse	CCGGCATGAACAACCATAGA
Glutathione S-transferase 4 (*gst*-4)	Forward	TTGCTGAGCCAATCCGTATC
	Reverse	GCTTCAGCTTTGACCATTCTTC
Glutathione-disulfide reductase (*gsr*-1)	Forward	CTCTCATCGTCTGTTCAATGGT
	Reverse	GAGTCCGACGGTTCCAATAAG
Prolyl carboxypeptidase (*pcp*-2)	Forward	CAGCTAGAGATGCCGATGTT
	Reverse	GTTGAAGGTGGTGCTTGATTC
Activating transcription factor 4 (*atf*-4)	Forward	GGACACATACAGCCACTTTCT
	Reverse	TCGCGATGACGTAAGATTGG
Glutamate-cysteine ligase (*gcs*-1)	Reverse	GTGGATGGCCCAAGAATACA
	Forward	GGAGTACCTGGATTCACATTCC

Primers for the genes *skn-1*, *ama-1*, *sod-1* were obtained from Integrated DNA Technologies (IDT, Coralville, IA, USA), while *gst-4*, *gsr-1*, *pcp-2*, *atf-4,* and *gcs-1* primers were obtained from Eton Biosciences, Inc. (Union, New Jersey, USA).
